# Temperature affects the silicate morphology in a diatom

**DOI:** 10.1038/srep11652

**Published:** 2015-06-26

**Authors:** N. Javaheri, R. Dries, A. Burson, L. J. Stal, P. M. A. Sloot, J. A. Kaandorp

**Affiliations:** 1 Computational Science, University of Amsterdam, Science Park 904, 1098 XH Amsterdam, The Netherlands; 2 FOM Institute AMOLF, Science Park 104, 1098 XG Amsterdam, The Netherlands; 3 Aquatic Microbiology, Institute for Biodiversity and Ecosystem Dynamics, University of Amsterdam, PO Box 94248, Amsterdam 1090 GE, The Netherlands; 4 Department of Marine Microbiology, Royal Netherlands Institute for Sea Research, PO Box 140, 4400 AC, Yerseke, The Netherlands; 5 ITMO University, St. Petersburg, Russian Federation; 6 Complexity Institute, NTU, Singapore

## Abstract

Silica deposition by diatoms, a common component of the phytoplankton, has attracted considerable interest given the importance in ecology and materials science. There has recently been a great deal of research into the biological control of biosilicifcation, yet the *in vivo* physical and chemical effects have not been quantitatively investigated. We have grown the marine diatom *Thalassiosira pseudonana* in batch culture at three temperatures (14^o^, 18^o^, and 23 ^°^C). We observed three distinct temperature-dependent growth phases. The morphology of silica was investigated using scanning electron microscopy followed by image analysis and supervised learning. The silica in the valves of the same species showed different structures: a mesh-like pattern in silicon-rich cultures and a tree-like pattern in silicon-limited cultures. Moreover, temperature affected this silica pattern, especially in silicon-limited cultures. We conclude that cells grown at 14 ^°^C and 18 ^°^C divide more successfully in Si-limited conditions by developing a tree-like pattern (lower silicification).

In their natural environment, phytoplankton communities are confronted with a variety of local and global changes including sunlight level, increasing temperature, acidity and nutrient concentrations. Phytoplankton communities may response differently to these changes but in order to survive and to be successful these organisms need to acclimate and eventually adapt adequately^1^,^2^,^3^,^4^. Diatoms (Bacillariophyta) represent one of the largest and most diverse groups of phytoplankton. In contrast to other phytoplankton groups, most diatoms are unique because they have a requirement for silicon, which is taken up as orthosilicic acid^5^,^6^. The silica in diatoms is deposited in a controlled way in a process called biomineralization^7^,^8^. The shell of the diatom made of amorphous hydrated silica is called a frustule. To prevent SiO_2_ dissolution of the diatom’s frustule in the ultra sub-saturated seawater, cells form an organic coat on their skeleton^9^. However, the regeneration of Si from dissolution of SiO_2_ (of mostly empty frustules) is a critical Si supply for diatoms in upper layers of oceans^10^,^11^. The rate of SiO_2_ dissolution depends on factors such as temperature, concentration of dissolved Si and activity of bacteria^9^,^11^,^12^,^13^,^14^,^15^.

One interesting example of an adaptation to environmental changes in diatoms occurs when the silicon supply is decreasing but other nutrients are in excess. In this situation, the cell cycle slows down, enabling maintenance of a slower growth rate rather than a cessation of growth^16^. Additionally, by slowing down the cell cycle diatoms may benefit from the dissolution of biogenic silicate from the frustules of neighboring individuals or a pulse of nutrient supply. Moreover, it has been observed that in a Si-limited environment the amount of silicification per cell also decreases resulting in thinner walls^16^,^17^. As a consequence, cell division could occur even under a low Si supply.

The silica deposition in diatoms is influenced by the nutrient availability in the medium and therefore by the dynamics of the cell population. Diatoms only divide when the daughter cells’ valves are synthesized and therefore cell cycle and growth of most diatoms is strictly controlled by the silicon availability^6^,^18^,^19^. As a result, after experiencing Si-starvation, the majority of diatom cells in a culture become synchronized via cells being stopped in their silicon sensitive part of the cell cycle. For instance, 60% to 80% of cells from *Thalassiosira pseudonana* species become synchronized after 24 h of Si-starvation^19^. The rates of silicon uptake and silica deposition in cells change through the cell cycle. Therefore, when studying cell level events like deposition of silica using population level data (such as silicon concentration in medium) one should consider that not all cells in one culture behave in the same way. Using the concept of an “average cell” might therefore introduce a significant error. For this reason, when studying population dynamics and cell dynamics the effect of non-synchronized cells should be taken into account^20^. Moreover, a variety of data analysis and mathematical modeling techniques have been developed for understanding the complex dynamics of cell populations^21^,^22^.

Understanding the biosilica morphology and the mechanisms controlling biosilicification is important from the material science perspective in addition to being crucial in silicon cycle of aquatic ecosystems. Ranging in size from a few micrometers to a few millimeters, diatoms develop structures in their silica shell in different orders of magnitude down to the nanometer scale. The frustule has two similar structures at the ends of the diatom, called valves. Valves are formed before the diatom divides. The rest of the silica is usually made of several bands, called girdle bands. The latter are formed while the cell is growing in size in several steps. The structure of the silica shell is species-specific and is therefore an important taxonomic characteristic. Silica formation in diatoms is mediated by biomolecules. For example, proteins such as sillaffin and silacidin and the long chain polyamines (LCPA) are known for their role in the organic matrix of diatoms’ biosilica^23^,^24^,^25^. In addition to biological control, there are physical and chemical mechanisms that mediate silicification. Examples of these are diffusion, nucleation, phase separation and self-assembly of silica nano-particles. Although there have recently been greater insights into the biological control via extracting and identifying biomolecules, the role of physical-chemical processes has been less investigated, especially *in vivo*. The physical-chemical effects can be examined by applying different environmental conditions to a single species of diatom.

Here, we study the effect of temperature and Si-limitation on silica morphology of individual cells from the same species *in vivo*. We use the cell size distribution of each population during the experiment as an indication of the different growth phases of the diatom culture. To quantify silica pattern differences, a new computational approach, including analysis of SEM images and classification algorithms, has been applied. The silica morphology is affected both by temperature and Si abundance. The results draw an overall picture of the changes in *Thalassiosira pseudonana* at individual cell and population levels due to differences in temperature and silicon availability.

## Results

We present results from an experiment with the diatom *Thalassiosira pseudonana* grown as batch cultures in incubators at temperatures of 14, 18 and 23 °C. We measured cell density and cell size distribution and investigated the morphology of the silicate deposits of the frustules. In all experiments the cell populations were large (>10^8^ cells) which provided a good representation of the possible effect of interaction of cells (a collective behavior).

### Cell density

**Figure 1:**
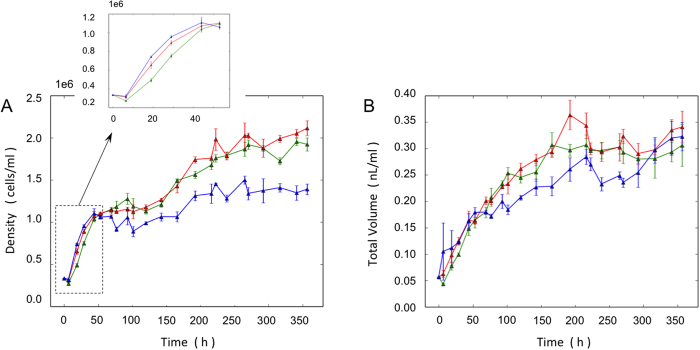
Growth of cells at different temperatures: 14 ^°^C (green), 18 ^°^C (red) and 23 ^°^C (blue). The mean and standard deviation is depicted. (**A**) Cell density. (**B**) Total cell volume per volume of seawater.

Figure 1A depicts the cell density of cultures during the observation time. In the first few time points, until 50 h (subplot in Fig. 1A), when the growth rate was maximum cultures grew fastest at 23 °C followed by those grown at 18 °C and 14 °C, respectively (the differences between the three temperatures is statistically significant up to 50 h and also after ~100 h until the end of experiment: p < 0.05 ANOVA). This is in agreement with the faster metabolism expected at higher temperatures. However, after 50 h, the 14 °C and 18 °C cultures grew faster than the culture grown at 23 °C. The lowest cell density was obtained in the cultures grown at 23 °C.

Figure 1B illustrates the population growth with the cell volume taken into account. All cultures showed similar growth in terms of total cell volume. Moreover, unlike the cell density curves, growth continued between approximately 50 h and 150 h.

### Cell size distribution and synchrony in population

**Figure 2:**
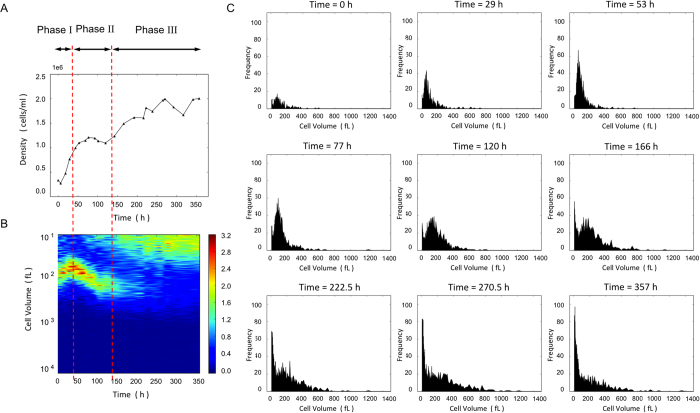
Cell density and cell size distribution of a culture grown at 14 ^°^C. (**A**) Cell density (**B**) Cell volume distribution. The color scale is the ratio of the number of cells with a specific volume compared to the total number of cells at each time point (measurement). The volume scale is logarithmic. The red lines separate three phases of growth. Non-limited Si/exponential growth phase, limited Si/synchronized phase and limited Si/regrowth phase. (**C**) Cell volume distribution of selected time points. In the first three frames increase in cell number is observed with the peak of cell volume distribution moving slightly to the left. In the 4^th^ and 5^th^ frame, the peak of the distribution moves to the right, meaning that most cells are growing in size. In the 6^th^ frame (166 h) the peak of the cell volume distribution breaks and a new peak over small volume cells appears. In the last frame (357 h) the large volume peak has almost disappeared and most cells seem to possess a small volume but in a large range of sizes.

For this experiment cells from a batch culture in late exponential phase were used. Cells in the Si-starved cultures were at least partially synchronized^26^,^27^. In all cultures three different growth phases were observed. The first phase was characterized by fast growth when all nutrients were in excess. The initial exponential growth slowed down when nutrients became depleted. During this phase, the initially partially synchronized culture at the end of the G1 part of the cell cycle starts from large cells that divide and become smaller. This is depicted in Fig. 2B. As shown in Figs 2A and 1A, the initial exponential growth gradually decreases until it reaches a plateau at the end of phase I.

Phase II starts when the cell density reaches a plateau. The beginning of phase II is defined by the time point at which the growth rate approaches zero. The silicon concentration is low at the onset of phase II. The Si, N and P concentrations were measured in the beginning of the experiment, at the end of phase I, and at the end of the experiment (Table S1). The girdle bands of the diatoms, which require the least silicon, form and thus the length of the cells (perpendicular to the valves plane) increases, progressing towards larger volumes during the later stages of phase II (Fig. 2B). In phase II, cells are synchronized and the difference in the size of biovolume is minimal. This is the reason for the continuous growth shown in Fig. 1B despite the number of cells was constant. Phase II cells are mostly of the same volume and in the same point of time in the cell cycle. During phase II the 24 h Si-starvation leads to synchronization of the cell cycle, a method that is widely used in diatom studies.

After approximately 100 h cells started to divide and the density increased again, transitioning the population in what we assign as growth phase III. The beginning of phase III was identified as the moment at which density changes between measurements exceeded 20%, which means a significant regrowth (Fig. 2).

At the end of the first phase the dissolved Si in the culture had greatly decreased and was deposited in the frustules. In order to continue growth, the organism can form daughter cells at a slower rate and eventually form smaller cells and survive (Fig. 2B). Alternatively, diatoms may dissolve some of the deposited silica and reuse that in order to obtain silicon for growth. It is known that diatom frustules dissolve in seawater. However, the silica dissolution rate of living cells is low and therefore most dissolved SiO_2_ will come from empty frustules^9^.

The cell size distribution of cultures grown at 18 ^°^C and 23 ^°^C followed roughly the same trend as that shown in Fig. 2 (Table S1). The cell volume distribution of all nine cultures and their three growth phases is shown in Fig. S1. Table 1 summarizes the time scales and densities of the three growth phases. In phase I, the time-scale of the initial fast growth, *τ*_exp_, was measured by fitting the data to 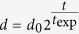 curve, where d_0_ is the initial density. Because the density is almost constant in the second phase, the average density during this phase, d_1_, is presented. In phase III the final density, d_2_ is shown. The values in table 1 illustrate that the cells grown in 23 ^°^C are the least successful in terms of cell density at the end of all phases, despite their initial growth time-scale being the shortest. In addition, the acidity of water was also monitored for all cultures. The pH values stayed in the range of 7.7 to 8.4 during the incubation (see Fig. S3).

### SEM and image analysis of the silicate morphology

**Figure 3:**
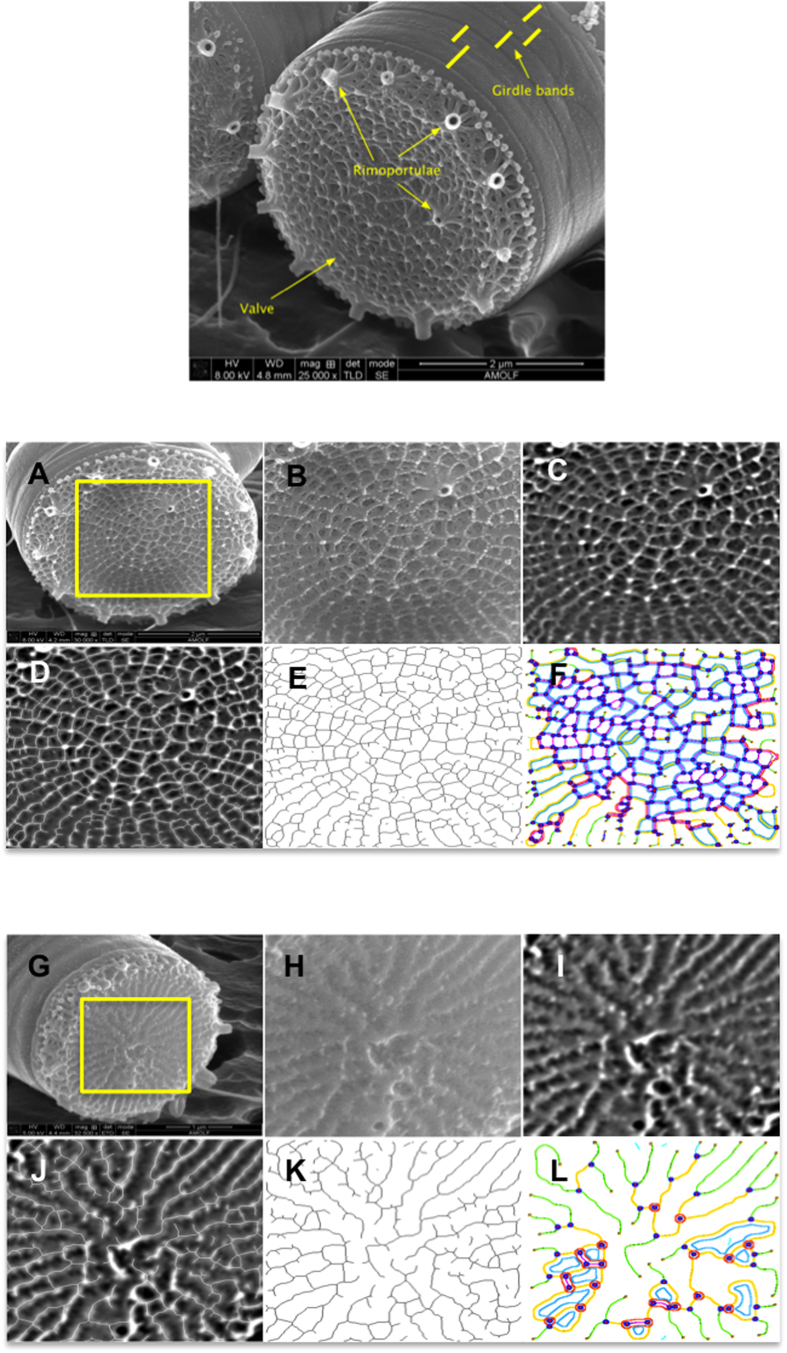
Analysis of silica morphology in diatom *Thalassiosira pseudonana.* (Top) A SEM image of the geometry of silica. Girdle bands are located on the sides. Valves are located at end of the cells. *Rimoportulae* are tube-like structures on the perimeter, and sometimes in the central area, of valves. (Middle and Bottom) Image analysis of the valve structure. Middle: Mesh-type pattern, Bottom: Tree-type pattern. (**A,G**) SEM image of a diatom (**B,H**) cropped area around the center of valve (**C,I**) after applying filtering too large and too small structures **(D,J**) after applying tree finding by Angiogenesis analyzer plugin (**E,K**) the extracted tree (**F,L**) after analyzing and quantifying the tree.

Figure 3 depicts the silica shell of the diatom *Thalassiosira pseudonana* from a scanning electron microscope recording. In addition to valves and girdle bands, in some diatoms such as in this species extra features exist such as the tube-like structure called *rimoportulae*, which is connected to the valves (Fig. 3).

We studied the effect of environmental conditions on the deposited silica structure of an identical species. This is specifically interesting since the diatoms in this experiment are all genetically similar and therefore the outcome can help us to understand the control mechanisms in silicification due to exterior influences. Samples for electron microscope imaging were prepared from all cultures at two time points. The first samples were taken at 29 h, when the cells were in their exponential growth phase (Fig. 1) and silicon was not limiting. The second sampled time point was at the end of the experiment (after 357 h) when cells experienced Si-starvation for a long time.

We used image analysis for measuring and comparing morphological properties in different types of patterns. First the “large features”, like valve diameter and number of *rimoportulae* was listed for each image. Second, for quantifying finer features such as number of nodes in the pattern, branch lengths and mesh area (enclosed areas of silica pattern) image analysis was performed on the valves’ patterns as a 2D structure. The features measured here were related to how the silica materials are located and how they intersect. In other words, a tree with branches that sometimes cross each other is considered. In addition, where the silica is surrounding an area, it is called a mesh area. Fig. 3 shows the image analysis steps to quantifying the branching or the mesh structure observed in the silica. The quantified features for each image of a diatom are listed in table S2. The definition of these features is based on the angiogenesis analyzer plugin for Image_J. After this step, SEM images are translated into a dataset with each row being an image (a diatom frustule) and each column being one of the 22 features in table S2.

### Classification

The SEM images of silica in the valves of *Thalassiosira pseudonana* show two distinct patterns. The first pattern (Fig. 3 - bottom) has branches radiating from the center and we refer to as “tree-like” pattern. The branches have been referred to as *silica ribs* in the literature [e.g. 24]. The second pattern (Fig. 3 - middle) has less branching and more connections and we refer to as “mesh-like” pattern. Most silica patterns are not entirely tree-like or mesh-like, but fall in between. Therefore, to quantify this property we use classification algorithms (supervised learning) to find the weight (probability) by which a pattern is a tree or a mesh. After classification, each image has some weight in the tree class with the remaining weight in the mesh class. The measured features have values in different scales. They therefore were all normalized to be in the range of zero to one.

In this study, three different learning algorithms were applied to the dataset: Decision Tree Classifier (DTC), Random Forest Classifier (RFC), extremely randomized tree or Extra Tree Classifier (ETC). For supervised learning a training set of data with defined class labels is provided in order to predict the class of the unlabeled data. The images that have an obvious tendency to the tree or mesh classes were selected as the training dataset. Since these methods have random elements in their calculations, each method was repeated 40 times. The internal parameters of the methods were selected such that the cross-validation, the measure of method’s accuracy, was at its maximum. The cross-validation test, which predicts the class of the labeled images, reveals the accuracy of the three classification methods. The RFC and the ETC methods are accurate (above 95%). We abandoned the DTC method due to its lower accuracy for the current dataset (see Fig. S4).

It is also informative to look at the importance of each feature in different classifiers for several runs of each method (Fig. S3). Even though the importance of the features is different in different methods and runs, still some information can be deduced. For instance, in neither of the two classifiers, valve diameter and number of *rimopotulae* per valve surface are important parameters in classification, suggesting that no correlation exists between ‘treeness’ or ‘meshiness’ for these features. In both methods, total mesh area and total branch length are important features in the classification.

**Figure 4:**
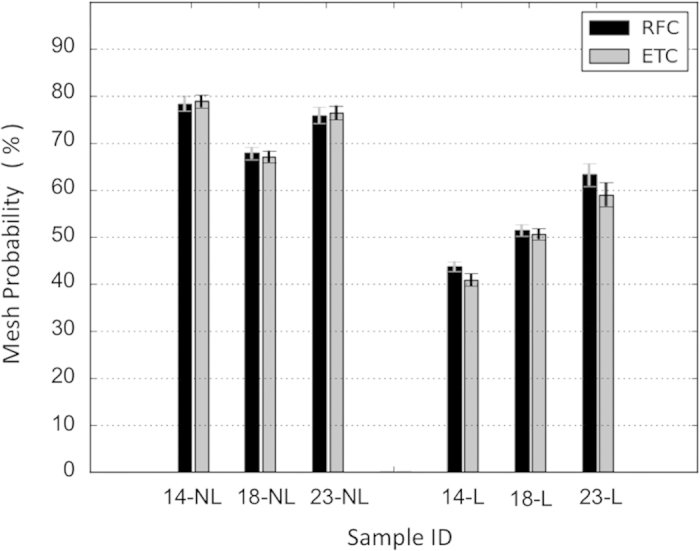
Mesh probabilities (meshiness) for each experimental sample. Cultures were grown at different temperatures (14, 18 or 23 degrees Celsius). Some are from a limited silicon population (L) and some are from a non-limited silicon population of diatoms (NL).

Finally, Fig. 4 represents the probability of a silica pattern from different experimental conditions to be mesh-like. Most importantly, diatoms grown under Si limited conditions show a much lower ‘meshiness’ than the non-limited young cultures. Temperature seems to affect the pattern of Si limited populations, whereas in Si non-limited cultures the temperature does not show a significant effect. In Si limited cultures, the ‘meshiness’ weight increases as the temperature rises.

## Discussion

The batch culture of *Thalassiosira pseudonana* exposed to different temperatures showed three different phases of growth. After reaching Si-limitation, a long period of not dividing (phase II of growth) followed by regrowth (phase III) was observed. The time-scale of the cell cycle during phase II and III was much longer than during initial exponential growth with abundant silicon. Moreover, temperature affects the population dynamics. The characteristics of the three growth phases were different at different temperatures. Cells grown at 23 ^°^C showed the highest initial exponential growth rate but the lowest final number of cells compared to the lower temperatures.

The silicification process in diatoms seems to be mediated by peptides and polyamines that have different forms in different species. In this study, although the experiments were done with only one species, we observed a variation in the morphology of valves. Two distinctive trends were observed in *Thalassiosira pseudonana*: tree-like and mesh-like structures. We noticed that the pattern of the frustules (the average over the population) showed significant changes after the physical and chemical factors changed. The silicon availability had a significant effect on the patterns. Old cultures, which were grown long-term in low silicic acid content showed more of the tree-type silica patterns on their valves. Younger cultures, which were still in the exponential growth phase with non-limited silicon supply, showed more of the mesh-type silica patterns. Previously, it was reported that diatoms show a diminished amount of silicification per cell under specific conditions. Under low silicification conditions the valves of *Thalassiosira pseudonana* show similarities with the tree-like pattern observed in this study^16^,^17^,^28^. It has been suggested that initially a *base layer* of silica containing the silica ribs is formed in the x-y plane and that additional silicification results in an extension in z-axis only in the direction of outside (distal surface). This model was suggested based on the detailed analysis of the structure of the silica in cells at different stages of growth^28^. The results presented in this paper challenge this model and we discuss this below using our statistical analysis of the silica structures developing under different conditions of growth according to equation 1.

We observed that temperature did not have a significant effect on the silica pattern or features of diatoms in the silicon-replete cultures (Fig. 4, NL cultures). However, in silicon depleted cultures temperature has a stronger effect on the pattern, such that the highest temperature had the highest mesh-like structure (Fig. 4, L cultures). To investigate this effect, one should take into account that the synthesized diatoms’ valves are passed to the next generations and therefore, in the Si limited cultures many of the valves on the live cells have been synthesized during the Si non-limited conditions. There is a possibility that the valves’ patterns change over generations due to dissolution of silica. But since the dissolution rate in the live cells is low and also the fact that only few generations have been proceeded during the time of this experiment, this possibility seems unlikely to have a big effect.

Furthermore, we can predict the expected mesh-like pattern percentage based on the cell density growth. For this purpose we assume that the valves that have been synthesized in the Si-replete conditions (phase I) retain their pattern (not any post-processing or dissolution of silica on live cells). We also assume that all the new valves that have been synthesized during Si-deplete conditions (phase II and III) have the tree structure with the mesh weight (meshiness) of mesh_p2p3_ = (1- mesh_p1_) in which mesh_p1_ is the mesh weight of the Si-replete culture. For instance, if we measure the meshiness of the population in non-limited silicon conditions and it is 80%, then we assume that meshiness of the valves synthesized during the limited silicon conditions is 20%. The final population at the end of the experiment contains valves both synthesized at Si limited and Si non-limited. Therefore, in order to calculate the meshiness of the final population under the mentioned assumptions, equation 1, which is a weighted average, is used.


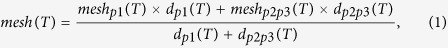


d_p1_ and d_p2p3_ are the culture density at the time of the first (29 h) and the second (357 h) SEM output respectively. Applying the values of density from Fig. 1A and the mesh weights of Si non-limited cultures from Fig. 4 to Equation 1, we calculate the mesh weight of Si limited cultures. The result is:

meshiness at 14 ^°^C = 43.4%

meshiness at 18 ^°^C = 47.6%

meshiness at 23 ^°^C = 60.5%

These values are in good agreement with the weight of Si limited cultures from the experiment (Fig. 4), which are 42%, 51% and 60% respectively. Therefore, the assumption that the silica patterns remained unchanged in our experiment and that most of the silica patterns synthesized in Si limited conditions are treelike is considered to be correct.

The presented results provide insight in the controlled process of the silica formation at nano-scales. The tree-like pattern synthesized at Si-limitation conditions could be simply due to the lower amount of silica nano-particles in the self-assembly process of silicification. If we assume that the “organic matrix” or the “organic soup of biomolecules” is the same for all the diatoms in this experiment, then the different supply of silicon can modify the equilibrium of the self-assembly and reaction-diffusion events and therefore generate a different pattern. It would be interesting to investigate this process further by a modeling approach. One example of the modeling of silica formation in diatoms was performed using diffusion limited aggregation (DLA) method^29^. This paper shows that at the lower supply of nutrient tree patterns are produced while at the higher supply of Si a more compact structure is formed. Moreover, using an agent-based model it was shown that a competition between diffusion and reaction in silicification results in different patterns^30^. This observation is relevant for the interpretation of the patterns we report in this study.

Our results show that the 23 ^°^C cultures are lower in density after experiencing Si limitation, revealing a lower rate of cell division and perhaps higher aggregation and mortality. In addition, there was little change in the patterns of silica in the Si-rich and Si-limited cultures grown at 23 ^°^C. Considering the results of the calculations using Eq. 1, we conclude that the effect of temperature on the silica pattern is mostly through changing the dynamics of the population. Moreover, the 23 ^°^C is less successful in dividing with the tree-like patterned valves under the Si-limiting conditions. This might be a disadvantage in the competitive conditions of aquatic ecosystems and especially in the light of the predicted increase of global ocean surface temperature.

## Methods

### Cultures and growth conditions

We grew the diatom *Thalassiosira pseudonana* in batch culture for about one week until silicon was depleted (less than 1 μmol.L^−1^). This culture was used as starter culture to inoculate 9 experimental batch cultures. Each flask was inoculated with 40 ml pre-culture in a total volume of 200 ml in TPP90301 flasks with filter cap. MDV medium was used with a silicon concentration of 100 μmol.L^−1^ (Text S1). Cultures were incubated in triplicate at 14, 18 and 23 ^°^C in illuminated incubators under an alternating light/dark regime of 16/8 h at a photon density of ~100 μmol m^−2^ s^−1^. The flasks were shaken when sampling in order to guarantee representative samples of the culture.

### Cell density and size distribution measurements

At each observation time point, the cell volume distribution of each culture was measured using a Beckman-Coulter-Multisizer 3 at 50-fold dilution with electrolyte. The integration of volume distribution was then used to calculate the cell density and the total volume of cells. In order to confirm the cell density measurements, cells were manually counted using a Bürker counting chamber and a light microscope at one time point. Moreover, the diameter and the length and, hence, the volume distributions were manually measured in SEM for a small number of cells (Fig. S2).

### Acidity measurement

The pH was measured directly in the samples by using a pH electrode and Knick Portamess pH meter.

### SEM samples preparation method

To prepare samples for SEM, we have used a protocol similar to^31^ with modifications. Briefly, the cells were collected by centrifugation (10 min, 500 rcf, Eppendorf centrifuge 5424R) from a 1.5 ml culture in microtubes and the supernatant was discarded. The cell pellet was re-suspended and fixed with 5% glutaraldehyde in 0.2 M cacodylate buffer for at least 3 h or overnight at 7 ^°^C. Cells were collected on a polycarbonate filter (Millipore, 25 mm diameter, 1 μm pore size, Whatman Nuclepore, WHA110610). The filters were washed twice in 0.1 M cacodylate, twice in 0.05 M cacodylate and eight times in MilliQ deionized water. Subsequently, the filters were dehydrated in a series of ethanol (30%, 50%, 70%, 80%, 95%, 100%) and then in hexamethyldisilazane (HMDS), ethanol mixture (1:1 v/v) and twice in 100% HMDS. The filters were stored in a desiccator. Before imaging with SEM, samples were coated with Chromium to 15 nm thickness using K575X Sputter Coater [Emitech] to minimize charging effects while imaging.

### Computational methods

SEM images were quantified using the Angiogenesis Analyzer plugin^32^,^33^,^34^ for ImageJ^35^. Fig. S4 shows the output of all SEM images after image analysis. For classification of the dataset, scikit-learn, a machine-learning library in python was used. Three supervised classification methods have been applied to the data: Decision Tree Classification (DTC)^36^,^37^, Random Forest Classification (RFC)^38^,^39^ and Extra Tree Classification (ETC)^40^.

## Additional Information

**How to cite this article**: Javaheri, N. *et al.* Temperature affects the silicate morphology in a diatom. *Sci. Rep.* **5**, 11652; doi: 10.1038/srep11652 (2015).

## Supplementary Material

Supplementary Information

## Figures and Tables

**Table 1:** Characteristics of three growth phases of diatom populations. The differences are statistically significant (p < 0.05).

**d**_**0**_** = 0.33 × 10**^**6**^	**Τ**_**exp**_ **: Doubling time-scale (h)**	**d**_**1**_ **: Average Density during phase 2 (cells/ml)**	**d**_**2**_**: Final density (cells/ml)**
14 °C	40.90 ± 4.46	(1.16 ± 0.03) × 10^6^ = 3.47 d_0_	(1.92 ± 0.08) × 10^6^ = 1.66 d_1_
18 °C	20.98 ± 1.84	(1.14 ± 0.03) × 10^6^ = 3.42 d_0_	(2.11 ± 0.09)×10^6^ = 1.85 d_1_
23 °C	17.25 ± 0.36	(1.02±0.02) × 10^6^ = 3.05 d_0_	(1.39±0.06)×10^6^ = 1.36 d_1_
ANOVA p-value	0.000307	0.003963	0.000204
