# Supplementary material for: Temperature affects the silicate morphology in a diatom
Source: Sci Rep. 2015 Jun 26;5:11652. doi: 10.1038/srep11652 (PMC4481522; doi:10.1038/srep11652)
Supplement: Supplementary Information [file srep11652-s1.pdf]

Supplementary Information:

Temperature affects the silicate morphology in a diatom

N. Javaheri[1], R. Dries[1&2], A. Burson[3], L.J. Stal[3&4], P.M.A. Sloot[1,5,6], J.A. Kaandorp[1*]

1- Computational Science, University of Amsterdam, Science Park 904, 1098 XH Amsterdam, The Netherlands
2- FOM Institute AMOLF, Science Park 104, 1098 XG Amsterdam, The Netherlands
3- Aquatic Microbiology, Institute for Biodiversity and Ecosystem Dynamics, University of Amsterdam, PO Box 94248, Amsterdam 1090 GE, The Netherlands
4- - Department of Marine Microbiology, Royal Netherlands Institute for Sea Research, PO Box 140, 4400 AC, Yerseke, The Netherlands
5- ITMO University, St. Petersburg, Russian Federation
6- Complexity Institute, NTU, Singapore

* Corresponding author: J.A.Kaandorp@uva.nl

Text S1. MDV Medium recipe for Diatoms

**minerals** (g ·l^{-1})	**conc. medium** (g ·l^{-1})	**conc. Stock**	**ml stock per medium per 1 l**
NaCl	24.1	241	100
Na_2SO_4	3.2	32	100
$MgCl_2 \cdot 6\,H_2O$	8.7	435	20
KCl	0.54	54	10
$CaCl_2 \cdot 2\,H_2O$	1.6	160	10

- Fill up to ca. **950 ml** with Aqua dest. and autoclave.
- For solid medium fill up to **400 ml** and sterilise agar (15g/l) separately in **550 ml** Aqua dest.

$NaHCO_3$	0.18	18	10
$NaNO_3$**	0.5	100	5
$NaH_2PO_4 \cdot H_2O$	0.0069	6.9	1
$Na_2SiO_3 \cdot 5\,H_2O$	0.0426	21.3	1
Citrate Mix	/	/	10
Trace Metal Mix	/	/	1
Vitamin 8 Mix	/	/	1
M2 (modified)	/	/	1
**or NH_4Cl	0.0268	26.8	1

- For liquid medium fill up to **100 ml** and add to the autoclaved medium through a sterile **0.2 µm filter.**
- For solid medium fill up to **50 ml.**

Citrate Mix:

	conc. Medium (g/L)	conc. Mix (g/L)
$C_6H_8O_7 \cdot H_2O$	0.003	0.3
Fe-NH_4-citrate	0.0036	0.36

Autoclave the Citrate Mix to avoid fungal and bacterial growth.

Trace Metal Mix:

conc. medium (g/L)	conc. stocks I (g/L)	**Trace metal mix** (ml/L)	
$CuSO_4 \cdot 5\,H_2O$	9.8e-6	9.8	1
$ZnSO_4 \cdot 7\,H_2O$	22e-6	22.0	1

$CoCl_2 \cdot 6 H_2O$	10e-6	10.0	1
$MnCl_2 \cdot 4 H_2O$	18e-6	18.0	1
$Na_2MoO_4 \cdot 2 H_2O$	6.3e-6	6.3	1
$Na_2SeO_3 \cdot 5 H_2O$	1.6e-9	0.016	0.1

Prepare apart a **stock** of each trace metal (**stock I**) and dilute 1:1000 for the final **Trace Metal Mix.**

M2 (modified):

**minerals**	**conc. medium ($g \cdot l^{-1}$)**	**conc. M2 ($g \cdot l^{-1}$)**	
KBr	0.039	39	modified
$SrCl_2 \cdot 6 H_2O$	0.010	10	modified
$AlCl_3 \cdot 6 H_2O$	0.000014	0.014	
LiCl	0.000003	0.003	
KI	0.00001	0.010	
H_3BO_3	0.011	11	modified
RbCl	0.00003	0.03	

Vitamin 8 Mix:

**vitamins**	**conc. medium ($g \cdot l^{-1}$)**	**conc. Stock ($g \cdot l^{-1}$)**	**stock I (g /100ml)**	**stock II (ml stock I / 100 ml)**
Biotin*	4e-8	0.00004	0.004	0.1
Thiamine-HCl	2e-5	0.02	0.02	10
Cyanocobalamin	8e-7	0.0008	0.08	0.1
Folic acid*	8e-8	0.00008	0.008	0.1
Inositol	2e-4	0.2	0.02	1
Nicotinic acid	4e-6	0.004	0.04	1
Thymine*	1.2e-4	0.12	0.012	1
Ca-d-pantothenate	4e-6	0.004	0.04	1

* Dissolve first in 1N NaOH and then add aqua dest.

Prepared 1000 ml of less vitamin solution and freezed in 1.1 ml portions (Eppendorfcaps) by - 20 °C and used as a reservoir for following media.

Table S1. List of nutrient content in seawater in three different time points of the experiment.

Temperature (°C)	Incubation time (h)	WNH4 (µmol N-NH4/L)	WNO2 (µmol N-NO2/L)	WNO3 (µmol N-NO3/L)	WNOX (µmol N-NOx/l)	WPO4 (µmol P-PO4/l)	WSi (µmol Si-SiO2/l)
Any	0	14	1.2	5764.8	5766	36	59.64
14	29	0	2.4	5958	5960,4	39,8	39,58
18	29	0	2.2	5971.4	5973.6	37.8	18.2
23	29	0	2.6	5945.4	5948	35.8	3.08
14	356	0	14	5453.2	5454.8	12.4	0.36
18	356	0	10.8	5399.6	5413.6	12.6	0.04
23	356	0	11.2	5518	5528.8	12	0

Table S2. List of features used in image quantification. Features 0-1 are geometry related. Features 2-21 are related to the nano-scale pattern, calculated from image analysis. Values of features 2-21 were divided by the analyzed area of the image to be comparable in different images.

0	Valve diameter	11	Number of segments
1	Number of Rimoportulae per valve surface	12	Number of branches
2	Number of Extremes	13	Number of isolated segments
3	Number of Nodes	14	Total length
4	Number of Junctions	15	Total branching length
5	Number of Master Junctions	16	Total segments length
6	Number of Master Segments	17	Total branches length
7	Total master segments length	18	Total isolated branches length
8	Number of meshes	19	Branching interval
9	Total meshes area	20	Mesh index
10	Number of pieces	21	Mean mesh size

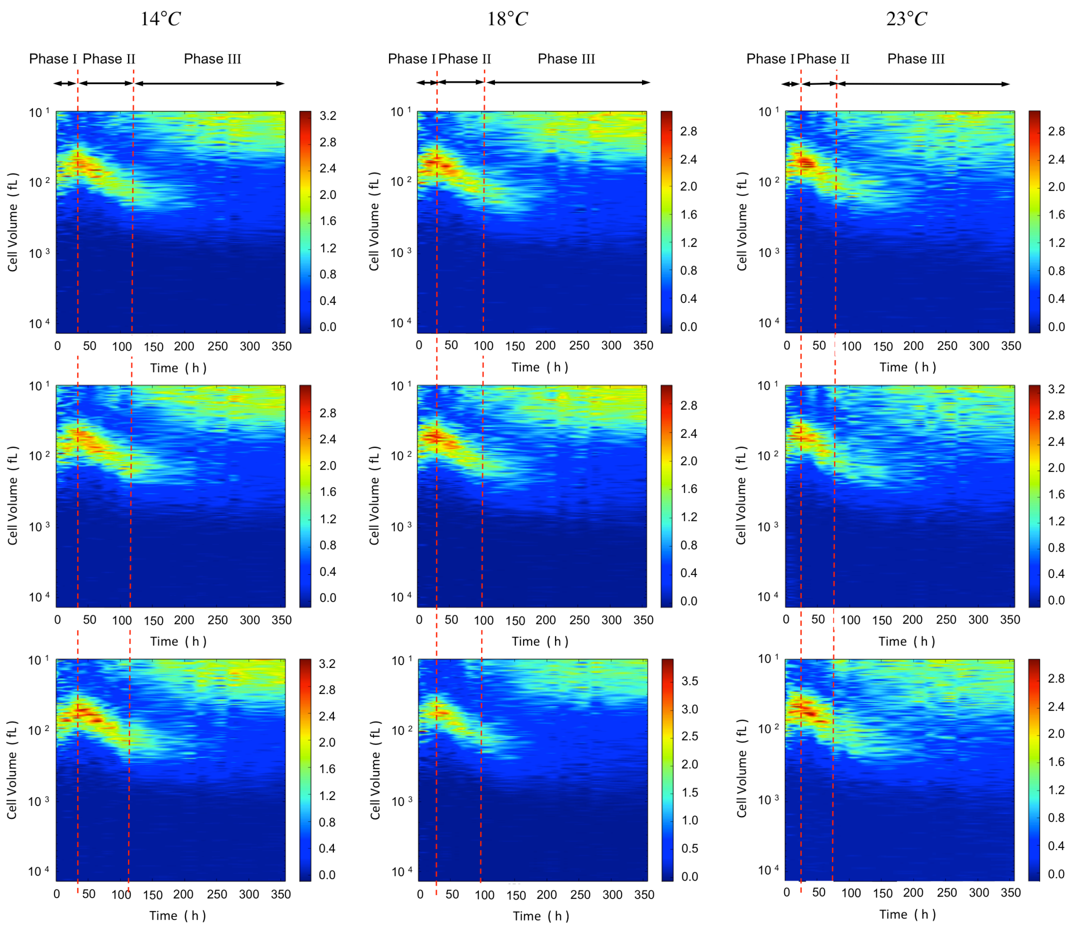

Fig. 1

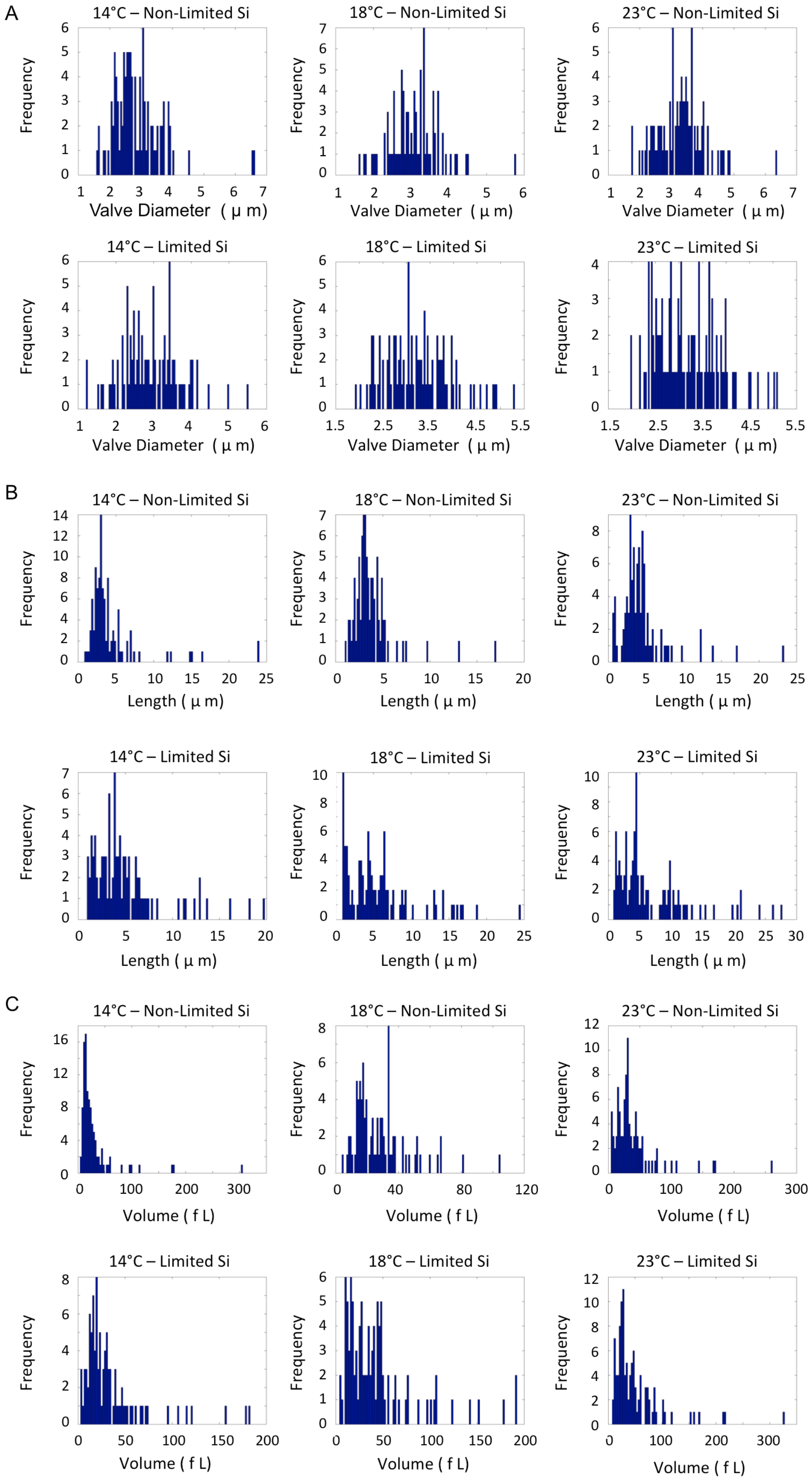
A
14°C – Non-Limited Si
18°C – Non-Limited Si
23°C – Non-Limited Si
Frequency
Valve Diameter (μ m)
14°C – Limited Si
18°C – Limited Si
23°C – Limited Si
B
Length (μ m)
C
Volume (f L)

Fig. 2

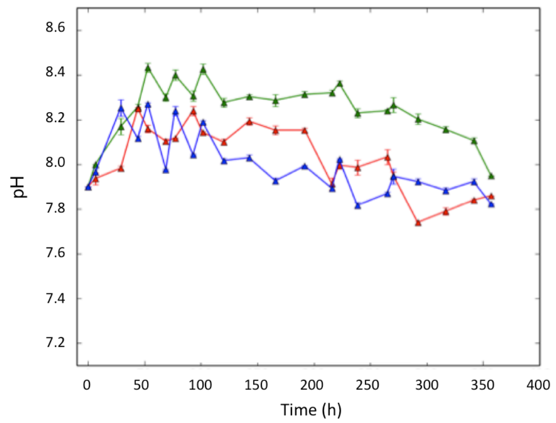

8.6
8.4
8.2
8.0
7.8
7.6
7.4
7.2
pH
0
50
100
150
200
250
300
350
400
Time (h)

Fig. 3

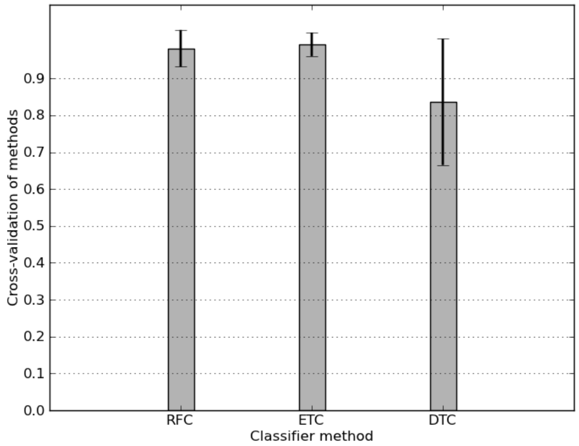

0.9
0.8
0.7
0.6
0.5
0.4
0.3
0.2
0.1
0.0
Cross-validation of methods
RFC
ETC
DTC
Classifier method

Fig. 4

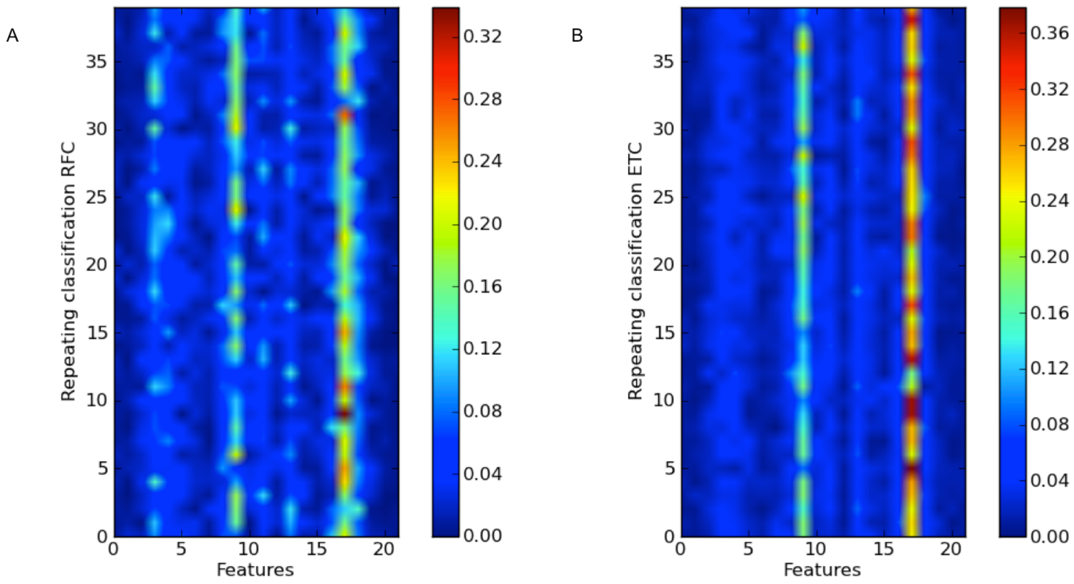
A
Repeating classification RFC
Features
B
Repeating classification ETC
Features

Fig. 5

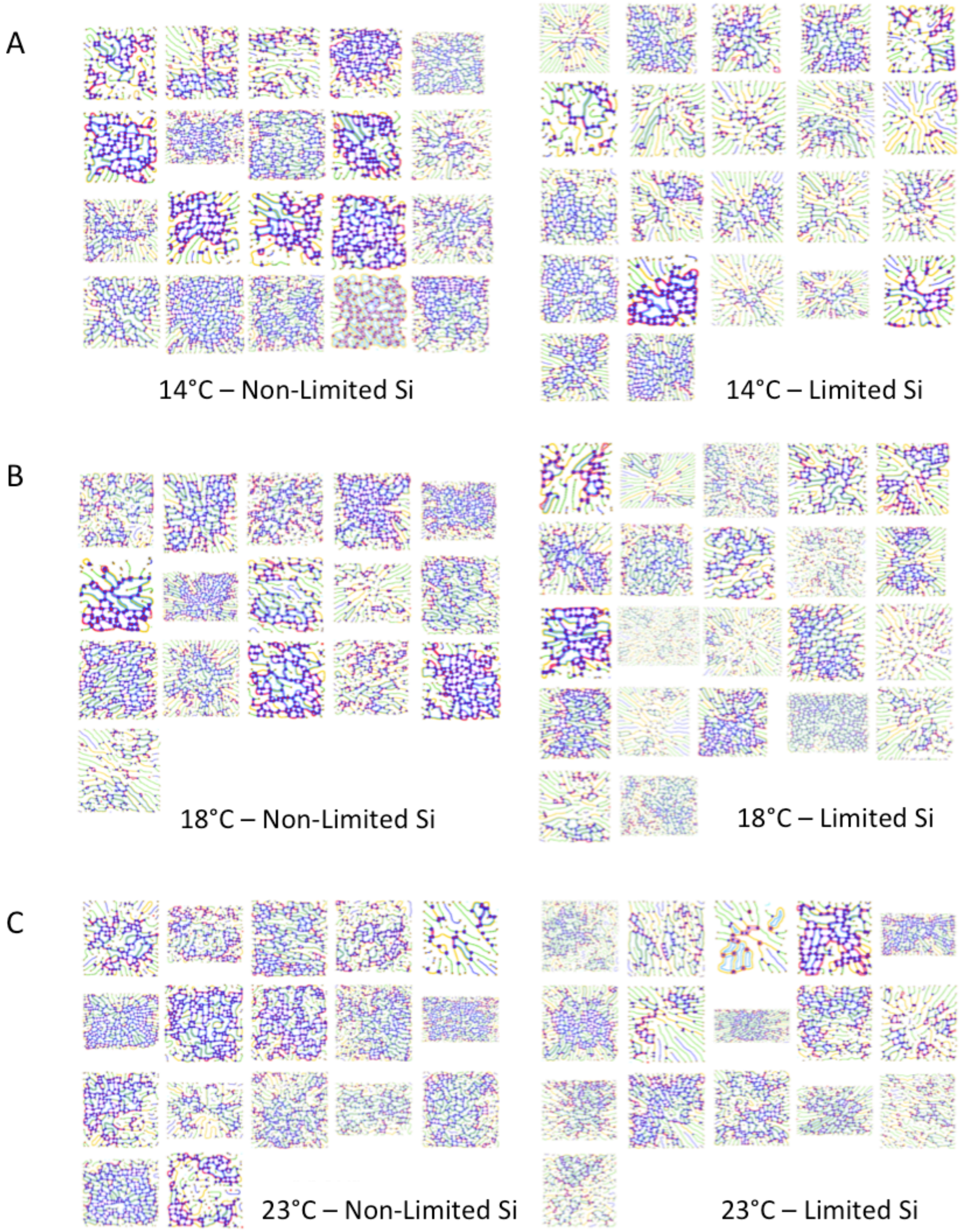
A
14°C – Non-Limited Si
14°C – Limited Si
B
18°C – Non-Limited Si
18°C – Limited Si
C
23°C – Non-Limited Si
23°C – Limited Si

Fig. 6

SI Figure Legends:

Fig. S1. Cell size distribution vs. time and growth phases – Three cultures was grown for each incubation temperature: 14, 18 and 23 degrees Celsius

Fig. S2. Diameter (A), length (B) and volume (C) distributions of diatoms grown in three different temperatures in limited or non-limited silicon samples. The measurements were performed manually using SEM images for approximately 100 cells in each sample.

Fig. S3. pH values measured for all 9 cultures. 14°C (green), 18°C (red) and 23°C (blue). For each temperature the mean and standard deviation values of three cultures are shown.

Fig. S4. Accuracy of the classification method using cross-validation test: The mean and the standard deviation values of each method over 40 runs are shown. DTC has the least accuracy for current dataset. RFC and ETC both show a very good accuracy.

Fig. S5. Feature importance in each classifier for 40 runs of the method. (A) Random Forest classifier (B) Extra Tree classifier

Fig. S6. Angiogenesis analysis of silica patterns of diatoms valves at different temperatures: 14(A), 18(B) and 23(C), for samples from cultures with non-limited silicon supply (left column) or limited silicon supply (right column).
